# MicroRNA-548 regulates high mobility group box 1 expression in patients with preterm birth and chorioamnionitis

**DOI:** 10.1038/s41598-019-56327-9

**Published:** 2019-12-24

**Authors:** Ga-Hyun Son, Youngmi Kim, Jae Jun Lee, Keun-Young Lee, Heejin Ham, Ji-Eun Song, Sung Taek Park, Young-Han Kim

**Affiliations:** 1 Division of Maternal-Fetal Medicine, Department of Obstetrics and Gynecology, Hallym University College of Medicine, Kangnam Sacred Heart Hospital, Seoul, Korea; 2 Yonsei University College of Medicine, Seoul, Korea; 3 Institute of New Frontier Research, College of Medicine, Hallym University, Chuncheon, Korea; 4 Departments of Anesthesiology and Pain Medicine, College of Medicine, Hallym University, Chuncheon, Korea; 5 Department of Obstetrics and Gynecology, Institute of Women’s Life Medical Science, Yonsei University College of Medicine, Seoul, Korea

**Keywords:** miRNAs, Translational research

## Abstract

High mobility group box 1 (HMGB1) is a prototypic alarmin and plays an important role in the pathogenesis of inflammatory process in spontaneous preterm birth. This study was conducted to compare the levels of HMGB1 in amniotic fluid and amnion membranes in women with chorioamnionitis/intra-amniotic inflammation to the levels in healthy controls. We also aimed to elucidate the involvement of microRNA-548 (miR-548) in regulating HMGB1 expression and its function in human amniotic epithelial cells (hAECs). A bioinformatics analysis predicted the binding of HMGB1 by the miR-548 cluster. A repressed and forced expression assay in hAECs was performed to investigate the causal relationship between the miR-548 cluster and HMGB1. The levels of HMGB1 in amniotic fluid and amnion membranes were significantly higher in patients with intra-amniotic inflammation/chorioamnionitis than in those without inflammation. The miR-548 was significantly under-expressed in amnion membranes from patients with chorioamnionitis than in normal term controls. Repressed expression of miR-548 up-regulated HMGB1 expression in hAECs and increased its release from hAECs. Moreover, forced expression of miR-548 suppressed HMGB1 and inflammatory cytokines in hAECs, which increased when treated with lipopolysaccharide. These results suggest miR-548 can alter the inflammatory responses in hAECs, and might be involved in the pathogenesis of preterm birth by regulating HMGB1.

## Introduction

During pregnancy, fetal chorioamniotic membranes are involved in a variety of physiological and pathological processes, such as accommodation and protection of the developing fetus, parturition, and response to intra-amniotic infections^1^. Infections and inflammations of the amniotic cavity are often accompanied by histologic chorioamnionitis, which is associated with both preterm delivery and adverse perinatal outcome^2^. Intra-amniotic inflammation (IAI) can be due to microbial invasion of the amniotic cavity or other mechanisms in which necrosis or cellular stress induces the release of mediators that activate the innate immune system^3–5^. Damage associated molecular patterns (DAMPs), including S100 calcium binding protein B (S100B)^6^, heat shock proteins^7^, interleukin (IL)-1α^7–9^, and high mobility group box 1 protein (HMGB1)^10,11^, are endogenous proinflammatory and pro-oxidative stress molecules that can induce the inflammatory process in patients without demonstrable infection (sterile inflammation)^7,10,11^. Acting through Toll-like receptor 2 and 4 (TLR2 and TLR4), and by the receptor for advanced glycation end products (RAGE), DAMPs recruit inflammatory cells, which in turn amplify the innate immune response, thus favoring cytokine activation^12^. It was reported that the RAGE-DAMP system is present in women with preterm birth and IAI. Activation of the RAGE-DAMP system correlates with the degree of inflammation and oxidative stress damage in amnion epithelial, decidual, and extravillous trophoblast cells^13^. In addition, many infectious and inflammatory diseases, particularly in early childhood, have a strong genetic predisposition. A substantial body of evidence has shown that there is genetic susceptibility in the duration of gestation and the risk of preterm birth^14–18^. Inherited differences in immune system genes influence the susceptibility to microbial infection, which is a well-known cause of acute chorioamnionitis^19–21^.

HMGB1 is a prototypic alarmin that has a central role in the pathogenesis of both sterile and infectious inflammation^22–29^. Elevated concentrations of HMGB1 may reflect engagement of DAMP-induced inflammation^26,27,29–31^. Although primarily located in the cell nucleus, HMGB1 can be secreted by innate immune cells in response to pathogenic products and can be released by injured or dying cells^32^. Buhimschi *et al*. reported that HMGB1 levels are increased in the amniotic fluid (AF) of women with IAI and preterm birth. Through explant experiments, the authors also showed that intra-amniotic HMGB1 might be released from the damaged amniochorion^33^. Additionally, intra-amniotic administration of HMGB1 was reported to induce spontaneous preterm labor and birth^34^. These findings suggested that intra-amniotic HMGB1 secreted from fetal membranes can induce premature labor and, therefore, may be involved in signaling parturition in the context of sterile intra-amniotic inflammation. The amnion is the innermost layer of the intra-amniotic cavity. Thus, we can postulate that the amnion, rather than the chorion, plays a major role in response to changes in the amniotic cavity, such as IAI^35^. However, little is known about the role of amnion in preterm birth determined by IAI.

MicroRNAs (miRNAs) are a class of non-coding small RNAs ranging from 18 to 25 nucleotides in size, which are involved in post-transcriptional regulation of gene expression by mRNA degradation or translational repression. The repression results from miRNA binding to the complementary sequence of the 3ʹ-untranslated region (3ʹUTR) of the target mRNA^36^. The roles of miRNAs are profound in various physiologic and pathologic processes, such as cellular growth, development, organogenesis, and apoptosis^37^. MiRNAs have been implicated in various disease states that include cancer and cardiovascular diseases, and are considered important therapeutic targets. In addition, miRNAs are involved in most types of inflammatory responses, and there is increasing evidence that miRNAs are effective in regulating the magnitude of inflammatory responses through their effects on cellular development and acute cellular function^38^. Despite the growing importance of miRNAs in pathogenic states, few studies have explored the role of miRNAs in IAI and their possible involvement in preterm birth. A recent microarray analysis compared the miRNAomes in plasma between patients with spontaneous preterm birth and those with delivery at term. A total of eight miRNAs were differentially expressed in preterm birth groups, and miR-302b, miR-1253, and a cluster of miR-548 miRNAs were significantly under-expressed in preterm birth cases compared to term controls^39^. However, the potential role of these miRNAs in preterm birth remains unknown.

Of the differentially expressed miRNAs in preterm birth groups, the miR-548 cluster and miR-302b were predicted to bind HMGB1 3′UTR by a bioinformatics analysis. Thus, we performed this study to (1) compare the levels of HMGB1 in amniotic fluid of patients with intra-amniotic inflammation to those in healthy controls, (2) compare HMGB1 expression in amnion membrane of patients with preterm birth and chorioamnionitis to normal term controls, and (3) investigate the expression of the miR-548 cluster in amnion membrane and explore the regulation of HMGB1 expression by the miR-548 cluster.

## Results

### HMGB1 expression in AF and amnion membrane

**Figure 1:**
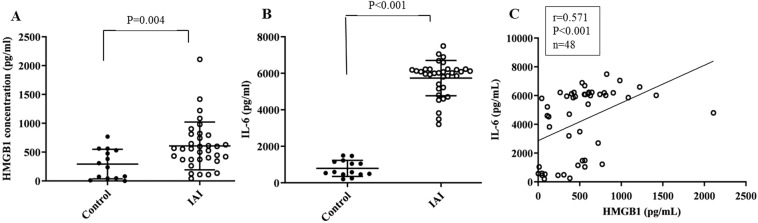
Levels of HMGB1 and IL-6 in amniotic fluid. (**A,B**) Levels of HMGB1 and IL-6 in amniotic fluid of women with and without intra-amniotic inflammation. (**C**) Relationship of amniotic fluid HMGB1 to the acute phase cytokine interleukin (IL)-6.

We analyzed HMGB1 levels in AF obtained from women with signs or symptoms of preterm birth who had amniocentesis to rule out infection (n = 38) or for either genetic indication (n = 10). Table 1 shows a comparison of clinical characteristics of patients with amniocentesis. Thirty four of 48 patients were diagnosed with IAI. The median [interquartile range] AF concentration of HMGB1 was significantly higher in patients with IAI than in those without IAI (563.8 [373.0–800.0] vs 273.0 [47.0–539.3] pg/mL, p = 0.004; Fig. 1A). The levels of HMGB1 significantly correlated with the levels of IL-6 (r = 0.571; p < 0.001; Fig. 1C).

**Table 1:** Clinical characteristics of patients with amniocentesis.

	Patients with IAI (n = 34)	Normal controls (n = 14)	p-value
Maternal age (years)	33 [30.8–36.0]	36.5 [33.8–38.5]	0.005
Previous preterm delivery	5 (14.7)	1 (7.1)	0.656
Gestational age at amniocentesis (weeks)	21.0 [19.6–22.4]	17.4 [16.7–19.9]	<0.001
Gestational age at delivery (weeks)	29.4 [21.0–38.0]	38.0 [37.0–38.5]	0.002
Maternal WBC count (cell/mL)	9,750 [8,375–12,350]	8,885 [7,395–11,012]	0.353
**Amniotic fluid analysis**			
HMGB1 (pg/mL)	563.8 [373.0–800.0]	273.0 [47.0–539.3]	0.004
IL-6 (pg/mL)	6,022 [5,199–6,224]	588 [465–1,175]	<0.001
Glucose < 20 mg/dL, n (%)	10 (29.4)	0	0.023
WBC (cells/mm^3^)	40 [7.0–137.5]	7.0 [2.0–9.3]	0.019
Positive cultures	2 (5.9)	0	1.000

Data presented as median [interquartile range] or n (%).

IAI, intra-amniotic inflammation; WBC, white blood cell; HMGB1, High mobility group box 1; IL-6, interleukin-6.

**Figure 2:**
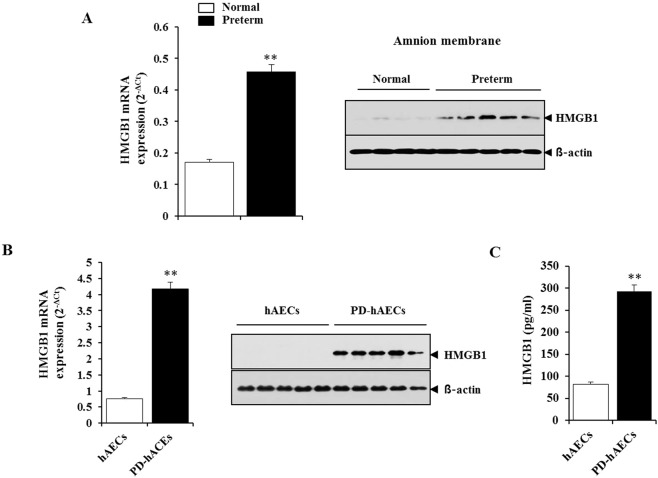
HMGB1 mRNA and protein expression in amnion membrane and human amniotic epithelial cells (hAECs). (**A**) HMGB1 mRNA and protein expression in the amnion membrane from women with preterm birth and chorioamnionitis and from normal term controls. (**B**) HMGB1 mRNA and protein expression in hAECs from patients with preterm birth and chorioamnionitis and from normal term controls. (**C**) HMGB1 levels in culture medium of hAECs from patients with preterm birth and chorioamnionitis and from normal term controls. **Significant difference (P < 0.001) compared with control group. hAECs, human amniotic epithelial cells; PD-hAECs, patient-derived human amniotic epithelial cells.

The expressions of HMGB1 mRNA and protein were increased in amnion membranes from women with preterm birth and acute chorioamnionitis as compared to normal controls (Fig. 2A). To confirm these data, we measured the expression level of HMGB1 in primary human amnion epithelial cells (hAECs) isolated from amnion membrane obtained from patients who delivered preterm due to acute chorioamnionitis (patient-derived hAECs, PD-hAECs) and women who had full-term pregnancy as normal control (hAECs). As expected, HMGB1 expression in PD-hAECs was significantly higher than in hAECs (Fig. 2B). Additionally, HMGB1 release from PD-hAECs was highly augmented compared to hAECs (Fig. 2C). These date implicated HMGB1 as a novel candidate biomarker for the early prediction of preterm birth determined by chorioamnionitis.

### MiR-548 cluster is under-expressed in amnion membrane from patients with preterm birth and acute chorioamnionitis

**Figure 3:**
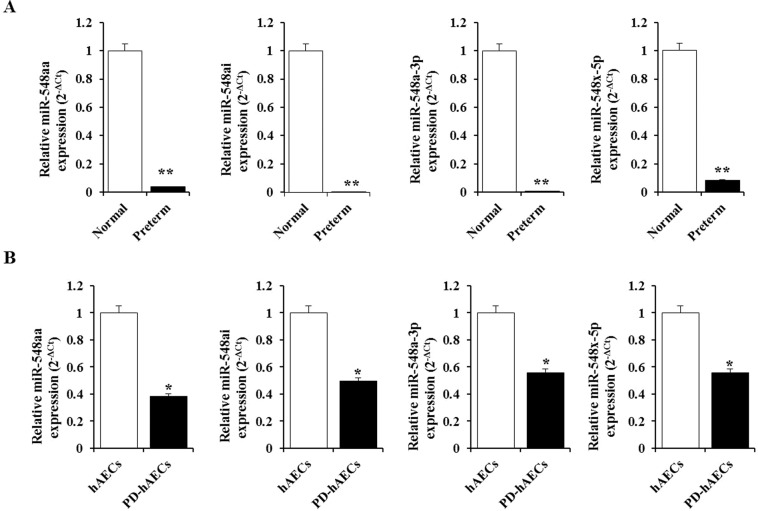
MicroRNA-548 cluster expression in amnion membrane and human amniotic epithelial cells (hAECs). (**A**) Levels of miRNA-548aa, miRNA-548ai, miRNA-548a-3p, and miRNA-548x-5p in amnion membranes from women with preterm birth and chorioamnionitis and from normal term controls. (**B**) Levels of miRNA-548aa, miRNA-548ai, miRNA-548a-3p, and miRNA-548x-5p in patient-derived hAECs (PD-hAECs) and hAECs from normal healthy women. **Significant difference (P < 0.01) compared with control group, *Significant difference (P < 0.05) compared with control group. hAECs, human amniotic epithelial cells.

Several miRNAs, including the miR-548 cluster, are reportedly downregulated in spontaneous preterm birth cases compared to controls^39^. Thus, we investigated the correlation between HMGB1 and the miR-548 cluster. Several binding sites of the miR-548 cluster were computationally predicted in the 3′UTR of HMGB1 (Table 2). To explore whether the miR-548 cluster could be a negative regulator of HMGB1 in preterm birth with acute chorioamnionitis, the expression level of the miR-548 cluster, including miR-548aa, miR-548ai, miR-548-3p, and miR-548x-5p, was measured using qPCR in the amnion membrane. Also, we investigated miR-302b, which displays reduced expression in spontaneous preterm birth^39^. As shown in Fig. 3A, all members of the miR-548 cluster were significantly decreased in amnion membranes from preterm women with acute chorioamnionitis compared to those from normal term controls. Furthermore, the expression of the miR-548 cluster was reduced in PD-hAECs than in hAECs from normal healthy women (Fig. 3B). However, there was no significant difference in the expression level of miR-302b between preterm and normal controls (data not shown). These results suggested that miR-548 cluster could be an inhibitory regulator of HMGB1 expression in preterm birth with acute chorioamnionitis.

**Table 2:** Predicted sequence alignment between miRNAs and HMGB1 3′UTR.

ID		Alignment	Position
miR-548aa	target (5′ → 3′) miRNA (3′ → 5′)		1298–1305
	target (5′ → 3′) miRNA (3′ → 5′)		1681–1686
	target (5′ → 3′) miRNA (3′ → 5′)		1499–1504
miR-548ai	target (5′ → 3′) miRNA (3′ → 5′)		1591–1596
miR-548a-3p	target (5′ → 3′) miRNA (3′ → 5′)		306–311
	target (5′ → 3′) miRNA (3′ → 5′)		2376–2381
	target (5′ → 3′) miRNA (3′ → 5′)		2024–2030
miR-548x-5p	target (5′ → 3′) miRNA (3′ → 5′)		523–529
	target (5′ → 3′) miRNA (3′ → 5′)		516–523
	target (5′ → 3′) miRNA (3′ → 5′)		869–874

### MiR-548 cluster can regulate expression of HMGB1 in hAECs

**Figure 4:**
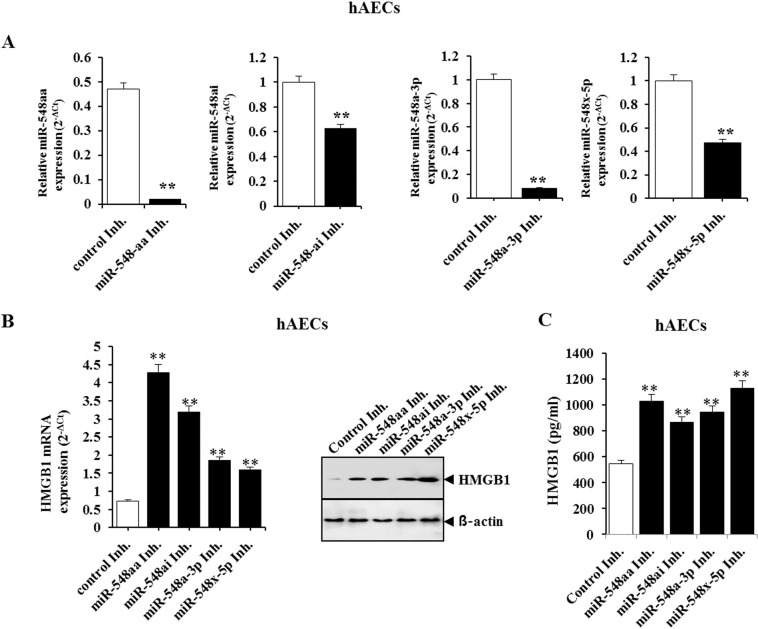
MicroRNA-548 cluster can regulate expression of HMBG1 in human amniotic epithelial cells (hAECs). (**A**) Levels of miRNA-548aa, miRNA-548ai, miRNA-548a-3p, and miRNA-548x-5p in hAECs transfected with miRNA-548aa inhibitor, miRNA-548ai inhibitor, miRNA-548a-3p inhibitor, miRNA-548x-5p inhibitor, and control inhibitor. (**B**) HMGB1 mRNA and protein expression in hAECs transfected with miRNA-548 inhibitor and with control inhibitor. (**C**) HMGB1 levels in culture medium of hAECs transfected with miRNA-548 inhibitor and with control inhibitor. **Significant difference (P < 0.01) compared with control group. hAECs, human amniotic epithelial cells.

To validate the correlation between HMGB1 and the miR-548 cluster in patients with preterm birth and acute chorioamnionitis, we investigated whether the expression level of the miR-548 cluster is crucial to the HMGB1 expression level using the synthetic mimics and inhibitors for the miR-548 cluster (Table 3) in hAECs. To examine whether the downregulation of miR-548 cluster could induce the enhancement of HMGB1 expression, hAECs were transfected with miR-548aa inhibitor, miR-548ai inhibitor, miR-548a-3p inhibitor, or miR-548x-5p inhibitor followed by qPCR (Fig. 4A). Inhibitors of the miR-548 cluster obviously increased the expression of both mRNA and protein levels of HMGB1 in hAECs compared to control inhibitor (Fig. 4B). Moreover, HMGB1 levels increased in the culture medium of hAECs transfected with miR-548 inhibitor compared to those transfected with control inhibitor (Fig. 4C). These findings indicated that downregulation of the miR-548 cluster contributed to the overexpression of HMGB1 expression in preterm birth with acute chorioamnionitis. In addition, the negative correlation between the expression of the miR-548 cluster and HMGB1 level was implicated in preterm birth with acute chorioamnionitis.

**Table 3:** Sequences of miR-mimic, miR-inhibitor, and controls.

Mimics or inhibitors	Sequence (5′ → 3′)	
**Mimics**		
control mimic	sense	UUCUCCGAACGUGUCACGUTT
	antisense	ACGUGACACGUUCGGAGAATT
miR-548aa mimic	sense	AAAAACCACAAUUACUUUUGCACCA
	antisense	GUGCAAAAGUAAUUGUGGUUUUUUU
miR-548ai mimic	sense	AAAGGUAAUUGCAGUUUUUCCC
	antisense	GAAAAACUGCAAUUACCUUUUU
miR-548a-3p mimic	sense	CAAAACUGGCAAUUACUUUUGC
	antisense	AAAAGUAAUUGCGAGUUUUACC
miR-548x-5p mimic	sense	UGCAAAAGUAAUUGCAGUUUUUG
	antisense	UAAAAACUGCAAUUACUUUC
**Inhibitors**		
miR-control inhibitor	CAGUACUUUUGUGUAGUACAA	
miR-548aa inhibitor	UGGUGCAAAAGUAAUUGUGGUUUUU	
miR-548ai inhibitor	GGGAAAAACUGCAAUUACCUUU	
miR-548a-3p inhibitor	GCAAAAGUAAUUGCCAGUUUUG	
miR-548x-5p inhibitor	CAAAAACUGCAAUUACUUUUGCA

### Lipopolysaccharide-induced inflammation induces enhanced HMGB1 expression through downregulation of miR-548 cluster

**Figure 5:**
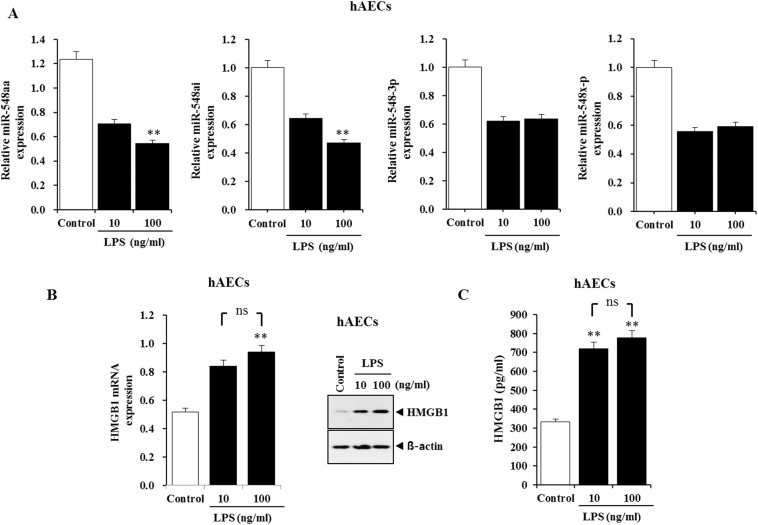
Lipopolysaccharide (LPS)-mediated inflammation induces enhanced HMGB1 expression through downregulation of microRNA-548 cluster. (**A**) Levels of miRNA-548aa, miRNA-548ai, miRNA-548a-3p, and miRNA-548x-5p in hAECs treated with 10 or 100 ng/mL LPS for 24 h. (**B**) HMGB1 mRNA and protein expression in hAECs treated with 10 or 100 ng/mL LPS for 24 h. (**C**) HMGB1 levels in culture medium of hAECs treated with 10 or 100 ng/mL LPS for 24 h. **Significant difference (P < 0.01) compared with control group, hAECs, human amniotic epithelial cells; LPS, Lipopolysaccharide.

To evaluate the role of the negative correlation between the miR-548 cluster and HMGB1 in preterm birth with acute chorioamnionitis, hAECs were treated with lipopolysaccharide (LPS) (10 or 100 ng/mL) for 24 h to induce inflammation followed by measurement of the expression levels of the miR-548 cluster and HMGB1. Compared to control, LPS inhibited the expression of the miR-548aa and miR-548ai and led to increases of HMGB1 mRNA and protein in hAECs (Fig. 5A,B). A significant elevation of HMGB1 levels was also observed in the culture medium of LPS-stimulated hACEs as compared to normal control medium (Fig. 5C). These findings indicated that downregulation of the miR-548 cluster promoted HMGB1 expression in hAECs and its subsequent release from the cells upon LPS-mediated inflammation.

### MiR-548 cluster can attenuate LPS-induced inflammation in hAECs

**Figure 6:**
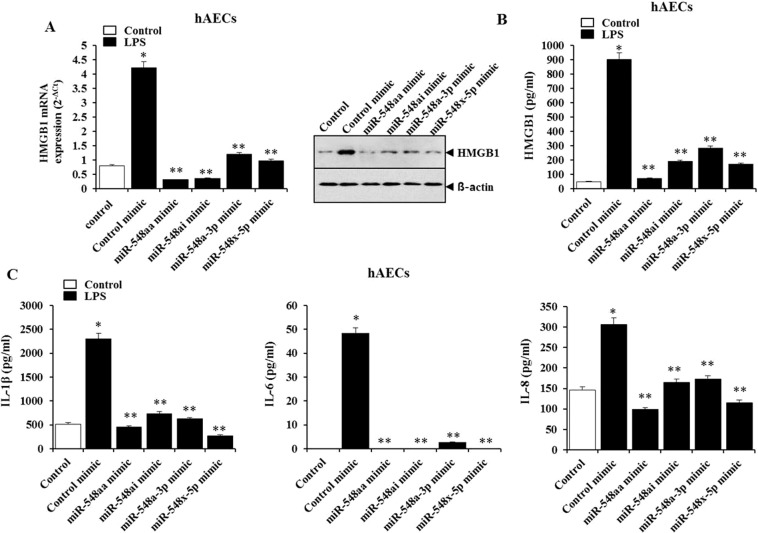
MicroRNA-548 cluster can attenuate lipopolysaccharide (LPS)-induced inflammation in hAECs. (**A**) HMGB1 mRNA and protein expression in miRNA-548 mimic and control mimic transfected hAECs that were not treated or treated with LPS. (**B**) HMGB1 levels in culture medium of miRNA-548 mimic and control mimic transfected hAECs that were not treated or treated with LPS. (**C**) IL-1β, IL-6, and IL-8 levels in culture medium of miRNA-548 mimic and control mimic transfected hAECs that were not treated or treated with LPS. **Significant difference (P < 0.001) compared with control mimic group, *Significant difference (P < 0.001) compared with control group. hAECs, human amniotic epithelial cells; LPS, Lipopolysaccharide; IL, interleukin.

To verify the effect of the miR-548 cluster on LPS-induced expression and release of HMGB1, hAECs were transfected with miR-548aa mimic, miR-548ai mimic, miR-548a-3p mimic, miR-548x-5p mimic, or control mimic. HMGB1 mRNA and protein expressions were pronounced in hAECs transfected with control mimic stimulated by LPS. In contrast, overexpression of the miR-548 cluster in cells transfected with miR-548 cluster mimics lowered HMGB1 induction upon LPS exposure (Fig. 6A). Moreover, HMGB1 levels were decreased in the culture medium of the miR-548 mimic transfected hAECs as compared to control mimic cells (Fig. 6B). Those findings indicated that the miR-548 mimic inhibited HMGB1 up-regulation in hAECs and the subsequent release of HMGB1 from hAECs exposed to an inflammatory stimulus. Furthermore, release of inflammatory cytokines (IL-1ß and IL-6) and chemokine (IL-8) was significantly reduced in the culture medium of hAECs transfected with miR-548 cluster mimics compared to control mimic cells. The data indicated that the overexpression of miR-548 cluster can suppress the inflammatory responses in hAECs.

## Discussion

This study demonstrates that the HMGB1 levels in AF from the patients with IAI were significantly higher than from those without IAI, and that HMGB1 was highly expressed in amnion membranes of patients with preterm birth and acute chorioamnionitis as compared to those in normal term controls. In addition, we validated that LPS-mediated inflammation could be responsible of the observed HMGB1 up-regulation in hAECs and for the release of HMGB1 from hAECs *in vitro*. Our findings, confirmed both *in vivo* and *in vitro*, were consistent with the fact the inflammation induced the expression of HMGB1 in the amnion membrane and its release from the amnion epithelial cells. HMGB1 is actively released by stimulation of the innate immune system with exogenous pathogen-derived molecules. Neurons, astrocytes, erythroleukemia cells, neuroblastoma cells, and other tumor cells are also reportedly able to actively secrete HMGB1^12,27–29,40^. Passive release of HMGB1 can be initiated when cellular integrity is damaged^32^. Considering that HMGB1 expression in hAECs increased when the cells were stimulated by LPS, HMGB1 could be released actively from amnion epithelial cells. Moreover, inflammation of the amnion membrane might lead to injury of amnion epithelial cells, which might cause the passive release of HMGB1. Regardless of the mechanism, the elevated levels of HMGB1 in AF could be considered a marker for amnion epithelial cell damage and injury, and representative of active signal transduction in amnion epithelial cells. It remains unclear how the released HMGB1 affects the amnion epithelial cells and plays a role in leading to preterm birth. We speculate that HMGB1 might induce cytokine, chemokine, and metalloproteinase synthesis that might be involved in preterm birth.

Little is known about the involvement of the miR-548 family in disease processes. Downstream targets of the miR-548 family are involved in breast cancer, inflammatory responses, and potential estrogen receptor sensitivity, indicating a potential biological role in the setting of preterm birth^41–43^. A recent study reported the suppression of endogenous miR-548 levels in the setting of viral infections^42^. Similarly, we observed that significant suppression of the miR-548 cluster was observed in the amnion membrane from patients with preterm birth and acute chorioamnionitis compared to normal term controls. Moreover, when hAECs were treated with LPS, miR-548 was downregulated. A previous study reported that inflammation does alter miRNAs at intestinal epithelial barriers and regulates intestinal permeability by degradation of tight junction protein, which is the target protein of miRNAs^44^. Furthermore, in cervical cells, inflammation can significantly alter the expression of specific miRNAs; these alterations might be involved in cervical remodeling and preterm birth^45^. Although the mechanism of miR-548 downregulation following inflammatory stimuli has not been clarified, our results suggest that under-expressed miR-548 can play an important role in acute chorioamnionitis determined preterm birth.

This study demonstrates that LPS downregulated the expression of the miR-548 in hAECs, and the inhibition of miR-548 induced the up-regulation of HMGB1 mRNA and protein expression, and release of HMGB1 in the culture medium of hAECs transfected with miR-548 cluster inhibitors. These findings suggest that IAI can induce downregulation of the miR-548 in the amnion membrane, thus leading to the up-regulated expression of HMGB1 in amnion and its release from amnion epithelial cells. Moreover, the miR-548 cluster mimic could reverse the LPS-induced up-regulation of HMGB1 in hAECs compared to control mimic. The release of HMGB1 from hAECs was also significantly decreased in hAECs transfected with miR-548 cluster mimics. In addition, the release of inflammatory cytokines and chemokine was suppressed in the culture medium of hAECs transfected with miR-548 cluster mimics compared to control mimic cells. These findings suggest that miR-548 cluster mimics can inhibit the up-regulation of HMGB1 that occurs when inflammation is stimulated, and alters inflammatory responses in hAECs. Thus, we can speculate that miR-548 could effectively reverse and prevent the activation of innate immunity, and significantly attenuate damage in IAI.

A key strength of this study is our demonstration that amniotic epithelial cells can release HMGB1. HMGB1 is increased in the AF of women with preterm birth and IAI. Further, increased AF HMGB1 can cause preterm birth. However, the source of AF HMGB1 had not been determined. Our findings identified the important role of the amnion membrane in preterm birth by releasing HMGB1 and other proinflammatory cytokines during IAI. In addition, we elucidated that there is a microRNA/target protein network involved in preterm birth with IAI/chorioamnionitis. A few studies have reported that there are miRNAs differentially expressed in women with preterm birth; however, little is known about the role of miRNAs in preterm birth and how their involvement may induce preterm birth. We have demonstrated that inflammation can alter miR-548 expression in the amnion membrane and suppression of miR-548 can enhance inflammatory responses in the AF, leading to preterm birth.

One limitation of this study is that we did not demonstrate direct binding of the miR-548 cluster to HMGB1. However, the bioinformatics data and our forced and repressed experiment adequately showed the relationship between miR-548 and HMGB1. In addition, we could not clearly determine whether a change in the amount of proinflammatory cytokines and chemokines secreted during LPS treatment was directly caused by altered miRNA expression or secondary to the secretion of HMGB1. Moreover, the gestational age of obtaining amniotic fluid and amnion could not be matched between the patient and control groups. Therefore, the difference in HMGB1 concentration between the patient and control groups may have been affected by differences in gestational age. Although, one previous study has reported no significant difference in the median AF HMGB1 concentration between women in the mid-trimester and those at term not in labor^11^, further studies are required to examine the HMGB1 concentration in AF and amnion membrane throughout pregnancy.

In conclusion, intra-amniotic inflammation induces the up-regulation of HMGB1 expression in the amnion membrane and HMGB1 release via suppression of miR-548. Moreover, miR-548 can attenuate inflammatory responses in amnion epithelial cells. Although the specific regulatory mechanism of miR-548 on HMGB1 expression remains unclear, these data demonstrate a potential molecular mechanism underlying the inhibitory effect of miR-548 on preterm birth, and implicate miR-548 as a potential therapeutic target in preterm birth with chorioamnionitis.

## Methods

### Patients and amniotic fluid collection

We investigated AF samples from 48 women with singleton pregnancies and clinically indicated amniocentesis. Samples were obtained by transabdominal amniocentesis for second trimester genetic karyotyping (n = 10) or to rule out AF infection in women who had preterm labor contractions refractory to tocolysis or advanced cervical dilatation (n = 38). Exclusion criteria were the presence of anhydramnios, human immunodeficiency or hepatitis viral infections, congenital anomalies, or abnormal karyotype. Gestational age was determined based on the last menstrual period confirmed by ultrasound examination prior to 14 weeks gestation. Preterm labor was defined as the presence of regular uterine contractions and documented cervical effacement and/or dilatation in patients before week 37 of gestation. IAI was defined by amniotic fluid interleukin (IL)-6 concentrations ≥2,600 pg/mL^46^. All women provided written informed consent before the collection of AF and placenta samples. The collection and utilization of the samples were approved by the Ethics Committee of Kangnam Sacred Heart Hospital (Ethics ref.: 2017-08-014). All experiments were performed in accordance with the relevant guidelines and regulations.

### Chemical and microbiological studies of AF

Following retrieval under sterile conditions, AF was analyzed for glucose concentration, white blood cell (WBC) count, gram stain, and for standard culture of aerobic and anaerobic bacteria, including Ureaplasma and Mycoplasma species. The remaining AF was aliquoted into 2-mL vials and cryopreserved at −80 °C.

### Enzyme-linked immunosorbent assay (ELISA) of AF HMGB1 and IL-6

The levels of IL-6 and HMGB1 in AF and cell culture medium (conditioned medium) were determined by an ELISA assay kit (Cusabio, Wuhan, China) according to the manufacturer’s instructions. The levels of IL-1ß and IL-8 in culture medium were determined by an ELISA assay kit (Cusabio and RayBiotech, Norcross, GA, USA, respectively) according to the manufacturer’s instructions.

### Amnion collection and processing

Placentas (n = 6) were obtained from women with suspected chorioamnionitis caused by prolonged latency after preterm premature rupture of membranes and/or preterm labor (median gestational age of 29.4 weeks, range 25.3–35.0 weeks), and from healthy women (n = 4) undergoing elective cesarean delivery at term in the absence of labor (median gestational age 38.1 weeks, range 20.1–39.0 weeks). Amnion membrane was manually stripped from the chorion and divided into small pieces (approximately 1.0 cm^3^ each). After sample collection, the remaining full-thickness placental biopsies were obtained and fixed in formalin. Biopsy specimens were interpreted by a placental pathologist who was blinded to the clinical and laboratory findings. Amnion samples histologically diagnosed with acute chorioamnionitis were used as patients’ samples in the experiments.

### Isolation of amniotic epithelial cells

Amnion membranes (n = 5, median gestational age 38.3 weeks, range 37.4–39.0 weeks) were obtained from women who had completed full-term pregnancy during elective cesarean deliveries before the onset of labor and from those (n = 5, median gestational age 23.3 weeks, range 18.3–34.5 weeks) with preterm birth and acute chorioamnionitis. The primary hAECs were isolated from freshly obtained amnion as described previously^47–50^. Briefly, the amnion membrane was washed with ice-cold phosphate buffered saline (PBS, pH 7.2) to remove blood clots and cellular debris, and cut into pieces approximately 6 cm long. The first enzymatic digestion was performed by incubating the membrane pieces with 20 mL of pre-warmed 0.05% trypsin/EDTA (Thermo Fisher Scientific, Waltham, MA, USA) at 37 °C for 10 min with gentle shaking. The cells obtained at this step were discarded to remove blood clots and cellular debris. Second and third digestions were conducted the same way. The membrane pieces were transferred into new 50-mL conical tubes containing 20 mL 0.05% trypsin/EDTA and incubated at 37 °C for 30 min with gentle shaking. The digestion mixtures obtained from the second and third digestions were filtered through a 70-μm cell strainer and centrifuged at 500 × g for 10 min. Cell pellets were suspended and grown in DMEM/F12 (Gibco, Franklin Lakes, NJ, USA) supplemented with 10% fetal bovine serum, 100 U/mL penicillin, 100 μg/mL streptomycin (Sigma-Aldrich, St. Louis, MO, USA), and 10 ng/mL epidermal growth factor (R&D Systems, Madison, WI, USA).

### Cell culture and reagents

The primary hAECs from amnion tissues were plated and maintained in culture at 37 °C in a humidified incubator in an atmosphere of 5% CO_2_. JetPrime transfection reagent was purchased from PolyPlus-Transfection (Strasbourg, France). Antibodies to HMGB1 (Cell Signaling Technology, Beverly, MA, USA) and Actin (Santa Cruz Biotechnology, Santa Cruz, CA, USA) were used. LPS from *Escherichia coli* strain 0111:B4 was obtained from Sigma-Aldrich.

### Western blot analysis

Western blot analysis was performed according to standard procedures^51^. Equal amounts of proteins were separated by 10% sodium dodecyl sulfate-polyacrylamide gel electrophoresis (SDS-PAGE) and transferred to polyvinylidene fluoride membranes (Thermo Fisher Scientific). Target proteins were detected by enhanced chemiluminescence reagents (Thermo Fisher Scientific).

### MicroRNA prediction of target genes and functional and bioinformatics analysis

Binding sites and sequences of miR-548 cluster on the HMGB1 3′UTR were predicted by the target prediction programs TargetScan (http://www.targetscan.org), MiRDB (http://mirdb.org), and miRmap (https://mirmap.ezlab.org). Paired sequence alignment is marked by red color and continuous lines (Table 2).

### RNA isolation and quantitative PCR (qPCR)

Total RNA samples were isolated from cells and amnion tissues using Trizol reagent (Invitrogen, Carlsbad, CA, USA). Five micrograms of RNA was used for cDNA synthesis using the Maxime RT PreMix Kit (iNtRON Biotechnology, Seoul, South Korea). PCR reactions were performed using a 2× Rotor-Gene SYBR Green PCR Master Mix (Qiagen, Carlsbad, CA, USA) in the Rotor-Gene Q (Qiagen). The primers used were HMGB1 (forward): 5′-ACATCCAAAATCTTGATCAGTTA-3′ and (reverse) -3′ (reverse) 5′-CTCCTTAATGTC ACGCACGA-3′; and Actin (forward): 5′-CATGTACGTTGCTA TCCAGGC-3′ (reverse) 5′-CTCCTTAATGTCACGCACGA-3′. For the analysis of miRNA expression, miRNAs were isolated from the hAECs and amnion tissues using miRNeasy (Qiagen), followed by reverse transcription with the miScript Transcription Kit (Qiagen). The miRNA expression level was measured with a miScript SYBR Green PCR Kit (Qiagen) using the Rotor-Gene Q (Qiagen). Primers for miRNAs and endogenous control U6 gene are shown in Table 4.

**Table 4:** Sequences of the primers used in real-time RT-PCR.

Designation	Sequence (5′ → 3′)
miR-548aa	AAAAACCACAATTACTTTTGCACCA
miR-548ai	AAAGGTAATTGCAGTTTTTCCC
miR-548a-3p	CAAAACTGGCAATTACTTTTGC
miR-548x-5p	TGCAAAAGTAATTGCAGTTTTTG
U6	CTCGCTTCGGCAGCACA

### Forced and repressed expression of miRNA-548 cluster

To verify the effect of miRNAs, miR-548 inhibitor, mimic, and negative control groups were transfected using JetPrime (Polyplus-Transfection). Total RNA, including miRNA and proteins, were isolated at 36 h post-transfection and were used for western blot analysis and qPCR. The sequences of miR-inhibitor and mimic are listed in Table 2. hAECs were incubated in the presence of 10 and 100 ng/mL LPS to induce inflammation. Culture medium was collected after 24 h of incubation to measure the levels of HMGB1.

### Statistical analyses

All experiments were repeated at least three separate times. Statistical analyses were performed using SPSS version 18.0 (SPSS Inc., Chicago, IL, USA). Data were compared with Student’s *t* test for parametric data or Mann-Whitney *U* test for nonparametric data. The Kruskal-Wallis test with Bonferroni corrections was used for multiple comparisons. Spearman correlations were used to measure co-linearity between the selected independent variables. Comparisons between proportions were done with Fisher’s exact test. Statistical analysis of the immunoassays data was performed after logarithmic transformation of data. Statistical significance was indicated when p < 0.05.
